# Calculation of Relative Binding Free Energy in the Water-Filled Active Site of Oligopeptide-Binding Protein A

**DOI:** 10.3390/molecules21040499

**Published:** 2016-04-15

**Authors:** Manuela Maurer, Stephanie B. A. de Beer, Chris Oostenbrink

**Affiliations:** Institute of Molecular Modeling and Simulation, University of Natural Resources and Life Sciences, Muthgasse 18, A-1190 Vienna, Austria; manuela.maurer@boku.ac.at (M.M.); stephanie.de-beer@boku.ac.at (S.B.A.d.B.)

**Keywords:** OppA, free-energy calculations, free-energy perturbation, molecular dynamics simulations, computational alchemy, thermodynamic integration, GROMOS

## Abstract

The periplasmic oligopeptide binding protein A (OppA) represents a well-known example of water-mediated protein-ligand interactions. Here, we perform free-energy calculations for three different ligands binding to OppA, using a thermodynamic integration approach. The tripeptide ligands share a high structural similarity (all have the sequence KXK), but their experimentally-determined binding free energies differ remarkably. Thermodynamic cycles were constructed for the ligands, and simulations conducted in the bound and (freely solvated) unbound states. In the unbound state, it was observed that the difference in conformational freedom between alanine and glycine leads to a surprisingly slow convergence, despite their chemical similarity. This could be overcome by increasing the softness parameter during alchemical transformations. Discrepancies remained in the bound state however, when comparing independent simulations of the three ligands. These difficulties could be traced to a slow relaxation of the water network within the active site. Fluctuations in the number of water molecules residing in the binding cavity occur mostly on a timescale larger than the simulation time along the alchemical path. After extensive simulations, relative binding free energies that were converged to within thermal noise could be obtained, which agree well with available experimental data.

## 1. Introduction

One of the most challenging tasks for computational chemists working in e.g., computational drug design is to predict not only whether active site water molecules are being replaced or conserved upon ligand binding, but to also quantify the thermodynamic characteristics of these processes [1,2,3]. The current study addresses these challenges regarding the role of water molecules in binding, using the periplasmic oligopeptide binding protein A (OppA) as a case study. We attempt to quantitatively reproduce ligand binding in this promiscuous protein in terms of relative binding free energies, using molecular dynamics simulations.

OppA is a part of the ATP-binding cassette import systems, one of the largest protein superfamilies known. It is one of the most abundant periplasmic proteins in e.g., *Salmonella typhimurium* and *Escherichia coli* [4]. It plays a crucial role in the transport of nutrients in Gram-negative bacteria, binding peptide fragments from the periplasm and shuttling them to a transmembrane transporter [5].

Bacteria are auxotroph for amino acids and OppA is, therefore, an important target for antibacterial drugs, potentially inhibiting bacterial growth for, e.g., *Escherichia coli*, *Salmonella typhimurium*, and *Borrelia* *burgdorferi* [6,7,8]. A different strategy than targeting growth directly is based on a “Trojan horse” principle, designing peptide-based antibiotics in such a way that they are bound and transported into the bacterial cell by OppA. OppA is known to bind some naturally-occurring toxic peptide analogues, e.g., the bacterial phytotoxin phaseolotoxin, which is a modified tripeptide produced by certain strains of Pseudomonas syringae [9]. Knowledge of OppA’s mechanism of substrate recognition, selectivity, and the role of water molecules in its binding process is of high interest to modern day drug design.

### 1.1. Ligand Binding and Promiscuity

Towards the end of the 1980s, the unusual character of OppA to bind various oligopeptides (rather than single specific amino acids or peptides of a fixed length) was discovered. OppA binds peptides of two to five residues regardless of composition and, therefore, has an immense amount of potential ligands [10]. It prefers positively-charged ligands, however, particularly lysine-containing tripeptides, due to the initial binding site’s negatively charged surface [11]. Upon binding, OppA goes through a large conformational change, which moves two domains towards the third as rigid bodies by ~26° in the crystal structures (with the open conformation being potentially even wider without crystal packing interactions) [12]. OppA’s closed conformation and its external appearance remains virtually identical though, masking the buried ligand’s individual size and charge.

The binding of OppA to a series of lysine-rich tripeptides was previously characterized experimentally by X-ray crystallography and isothermal titration calorimetry (ITC) [13,14,15]. One striking observation from these experiments was that the protein seems very promiscuous towards the central amino acid of a tripeptide: Keeping the first and third residue constant (lysine) while changing only the second residue in the center position (X = varying amino acids, 20 natural and eight non-natural), OppA bound all 28 investigated tripeptides (KXK), showing a rather wide range of binding affinities. However, these binding affinities seem strikingly uncorrelated with the nature (polar, apolar, aromatic, or charged) or size of the central residue.

### 1.2. Crystal Structure and Water

Subpocket 1 of OppA accomodates the N-terminal lysine of the peptide ligands. A salt bridge between the N-terminal nitrogen and the protein contributes to unspecific peptide binding, and a second salt bridge or hydrogen bond between side chain nitrogen and the protein contributes to OppA’s preference for lysine.

**Figure 1:**
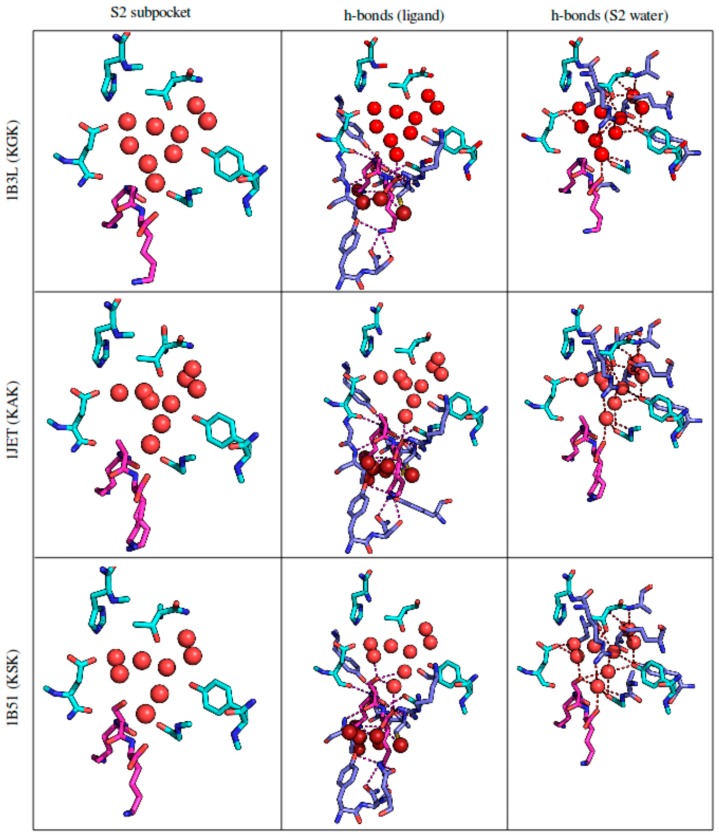
The S2 binding pocket of OppA, taken from the respective crystal structures. Protein residues pointed out by reference [15] are colored in cyan. Protein residues other than these that make hydrogen bonds with either the ligand or S2 subpocket water are depicted in lavender blue. The tripeptide ligand and its hydrogen bonds are colored in magenta. Water molecules are depicted as spheres: Water in the S2 subpocket (pointed out by reference [15]) and its hydrogen bonds are colored bright red; water with bonds to the ligand is colored dark red. Hydrogen bonds were estimated by PyMol’s polar contacts function, which is based on DSSP. For details on individual water molecules see Figure S1 in the Supplementary Materials.

Subpocket 3 accomodates the C-terminal lysine of the ligand. Again, a salt bridge between the ligand backbone (carboxylic acid) and the protein contributes to unspecific peptide binding. The lysine side chain in this position only makes a hydrogen bond however; it is more flexible and disordered than lysine 1, which can be observed as two conformations in the crystal structure of the KAK-OppA complex depicted in Figure 1. Lys-3 was found to contribute little to binding in general, in agreement with OppA’s ability to bind peptides of other lengths than three residues [16]. Even a tripeptide representing a naked backbone (GGG) is bound by OppA with small but measurable affinity [15].

Subpocket 2 is filled with conserved water molecules. OppA uses water for three distinct purposes: filling space not occupied by smaller ligands, satisfying hydrogen bonds of both ligand and host, and dissipating charges on the buried ligand [10]. The protein is evolutionarily optimized for its role as a nutrient delivery vehicle, utilizing water to its utmost efficiency.

Like in a number of other known periplasmic binding proteins (e.g., the arabinose-binding protein), OppA’s rigid domains are connected by a flexible hinge that closes upon ligand binding in a “venus flytrap” mechanism, completely enclosing even highly charged ligands along with water in the protein interior [12]. Some tightly held water molecules are an integral structural component of the binding surface, and some become buried with the ligand [10]. These buried water molecules are crystallographically sharply defined, and highly conserved between the differently ligated (KXK series) crystal structures [15].

Water surrounds and solvates all ligand side chains such that they make few direct interactions with the protein. The backbone moiety however shows several interactions with the protein. Figure 1 highlights the two different hydrogen bonding networks—one involving the ligand and the second involving the active site waters—and their separation in space. Only a single conserved water molecule (Number 5, see Figure S1, Supplementary Materials) participates in both networks.

The total potentially available binding pocket volume in OppA has been estimated at an enormous ~1000 Å^3^ [17]. Nevertheless, the binding mode geometry of all ligands in the published KXK series is virtually identical, with the backbone occupying a single specific pose in extended conformation, and the side chains being solvated in active site water. In spite of this common binding mode, the observed binding affinities vary over a relatively wide range. As direct interactions of either backbone or side chains with the protein cannot be the cause of observed differences in binding affinity for the KXK series, it is suspected that the different ligands are indirectly distinguished by their slightly different disruption of the conserved binding pocket solvent network. In this way, otherwise chemically highly similar ligands (such as KEK and KDK [15]) displace a different amount of water molecules from their optimized, orderly position. Just a small increase in ligand size causes a disturbance of the precarious balance between enthalpy of binding and entropy of water release.

### 1.3. Thermodynamic Contributions to Binding

Although ligand binding to OppA is determined by ITC measurements to be entropy-driven, neither the entropy change ΔS nor the enthalpy change ΔH show any correlation with binding affinity [15]. A strong entropy-enthalpy compensation complicates attempts to analyze binding contributions in the OppA system [14,15].

A study trying to derive an OppA-specific model for quantitative structure-activity-relationships (QSAR) successfully correlated model-predicted ΔS and ΔH values with their experimental counterparts, but failed for the combination of both into ΔG as well as for a separate independent ΔG model [18]. Note that the KGK ligand that is also investigated in the present study was considered to be an outlier due to its exceptional conformational freedom.

More recently, using a hybrid quantum mechanical/molecular mechanical calculation method for direct non-bonded interaction energies (to estimate binding enthalpy), and a semi-empirical Poisson-Boltzmann/surface area method to account for desolvation effects, a correlation of each of these energies to the experimental binding affinities could not be established [16]. Only the sum of these estimates showed a weak correlation with affinity, confirming again that both enthalpic and entropic effects (e.g., desolvation) must be taken into account just to qualitatively predict relative binding free energies.

### 1.4. Scope

In this study, we explore the binding of selected tripeptide ligands to OppA from *Salmonella typhimurium* [15]. The three ligands considered are KGK, KAK, and KSK, and their dissociation constants K_d_ and enthalpy changes ΔH were directly measured via ITC [15]. Changes in Gibbs binding free energy ΔG and in entropy ΔS were derived from these measurements. Binding free energies are given in Table S1 in the Supplementary Materials.

The three investigated ligands were chosen from the available ones due to their structural similarity but significantly differing ΔG_bind,exp_. The ligands, plus the residues lining the S2 subpocket which accommodates the X residue of KXK, are depicted in Figure 1. A superposition of the three crystal structures used in this study, including a closer look on the conserved water molecules in the S2 subpocket, can be found in Figure S1 in the Supplementary Materials.

It remains difficult to incorporate solvation effects at a high level of computational accuracy. Consequently, computational tools often neglect them, despite their great importance for modeling studies [19]. Quantitatively reproducing the experimentally determined binding affinities of the OppA-KXK complexes for example has, to the best of our knowledge, not been reported so far, even though the experimental characterization, *i.e.*, the crystallographic and calorimetric information, was already published in the mid-1990s.

Previous attempts to describe the system involved either empirical correlations based on 3D-QSAR [18], and implicit solvent calculations [16], ignoring many entropic aspects, or used a coarse grained model to qualitatively asses (not quantify) the system [17]. Both approaches lose the atomistic detail of individual water molecules by definition. Correlating binding affinity with structure by an empirical scoring function or 3D-QSAR either succeeded in qualitative predictions but was not quantitative [16], or failed to qualitatively reproduce the observed ligand ranking [14].

In our opinion, a successful calculation on the challenging OppA system can only be performed by a method that explicitly takes the individual interfacial water molecules that fill up the active site into account. Furthermore, as ligand binding in this case is known to be entropy-driven [15], flexibility of the protein and peptide ligands has to be taken into account explicitly as well. Hence, molecular dynamics (MD) simulations, together with the underlying statistical mechanics, is the method of choice to study tripeptide binding to OppA.

However, due to the large conformational change that OppA is known to undergo upon ligand binding, directly simulating the whole binding process seems an inexpedient approach. Instead, differences in free energy of binding between the three chosen ligands can be more advantageously calculated by mutating the ligand *in situ*. By comparing minimal alchemical ligand mutations in bound and unbound states, their relative binding free energy can be conveniently estimated by sampling relevant conformations and extracting the appropriate ensemble averages (see Methods below). This is a rather computationally-intense, but rigorous, approach which allows us to correctly incorporate the effects of active site water molecules on the binding thermodynamics.

## 2. Materials and Methods

### 2.1. Simulation Settings

All molecular dynamics simulations were performed using the GROMOS11 biomolecular simulation package [20]. Interaction parameters to describe the tripeptide ligands and proteins were taken from the GROMOS parameter set 45A3 [21].

All protein simulations were run using the crystal structure of OppA in complex with either KGK (PDB code 1B3L, 2 Å resolution) [15], KAK (PDB code 1JET, 1.2 Å resolution) [10], or KSK (PDB code 1B51, 1.8 Å resolution) [15] as initial structures. Starting configurations of tripeptide ligands in the freely-solvated, unbound simulations were directly extracted from their respective crystal structures. The ligands and protein-ligand complexes were solvated in periodic rectangular boxes, containing ~2000 and ~15,500 SPC water molecules [22] respectively, using a minimum solute-to-wall distance of 0.8 nm.

In all MD simulations, a time step of 2 fs was used. In an initial equilibration phase, the system was gradually heated up to 298 K, in three (water) and seven (protein) separate simulation steps of 20 ps each. In the first six protein equilibration steps, position restraints were applied on the protein and ligand atoms, using a gradually decreasing force constant (starting from 2.5 × 10^4^ kJ·mol^−1^·nm^−2^). Covalent bonds were constrained using the SHAKE algorithm [23], with a relative tolerance of 10^−4^.

During the production simulations, temperature (298 K) and pressure (1 atm) were kept constant by using the weak coupling algorithm [24], with coupling times of 0.1 ps and 0.5 ps, respectively. Solute and solvent degrees of freedom were coupled to separate temperature baths. The isothermal compressibility was set to 4.575 × 10^−4^ (kJ·mol^−1^·nm^−3^)^−1^. At every time step, non-bonded interactions were calculated up to a distance of 0.8 nm, by means of a pairlist that was generated every five steps. At every fifth time step, intermediate-range interactions at distances up to 1.4 nm were calculated and kept constant between updates. To account for electrostatic interactions beyond the cut-off radius of 1.4 nm, a reaction-field contribution was added to the energies and forces [25], with a dielectric permittivity of 61 [26]. During the simulations, coordinates were stored every 250 steps (0.5 ps). Protein stability during the simulations was assessed via monitoring of backbone RMSD and was found to be unobtrusive (data not shown).

Due to a surprisingly large hysteresis even for the small tripeptide system free in aqueous environment, the commonly used initial softness parameter settings of α_VdW_ = 0.5 and α_CRF_ = 0.5 nm^2^ in our preliminary studies (data not shown) were increased to 1.0 and 1.0 nm^2^, respectively. This change avoids unfeasibly long sampling times to achieve convergence. All calculations described in the present study employ the increased softness parameters, except when explicitly stated otherwise.

The softcore potential is used to adjust the high repulsive energies resulting from overlapping atoms during an alchemical transformation [27]. It is thus most relevant for the transformation of non-interacting dummy atoms into their regularly interacting equivalent, and irrelevant in the end states described by the pure Hamiltonians of A and B (see Section 2.3). TI curves for alchemical transformations may differ in their curvature for intermediate λ-points (see Section 2.3), but the integrated free-energy differences are unaffected. The effect of different parameter settings on calculation efficiency have been investigated before [28,29] and will be studied in more detail for the current system in future work.

### 2.2. Simulation Scheme

**Figure 2:**
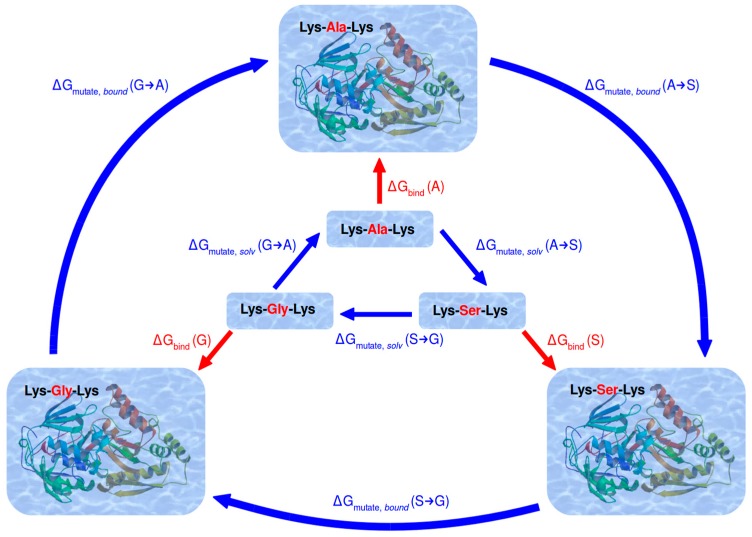
Thermodynamic cycle constructed between the three investigated ligands of OppA to calculate their relative binding free energy. Physical states and processes are denoted in red, whereas alchemical processes along unphysical intermediate states are denoted in blue.

Alchemical transformations were calculated between all three possible pairs of the three chosen ligands, as depicted in Figure 2. Detailed information on and schematics of the transformation process can be found in Figures S2 and S3 in the Supplementary Materials.

In water environment, each transformation was carried out twice, starting from either of the two end states (*i.e.*, ligands extracted from their respective protein crystal structure). In the complexed protein environment, each ligand transformation was carried out four times: once starting from either of the two crystal structures (*i.e.*, forward calculation), and once starting from the final output structure (after the last equilibration step) of a previously completed transformation (see Figure S2 in the Supplementary Materials). This scheme resulted in twelve calculations in total for the ligand-OppA complexes. These independent sets of calculations were performed in order to properly consider the distinct solvation patterns, and to provide replicates of each simulation.

For one alchemical transformation, always at least 11 equidistant λ-values were used. After an equilibration time of 50 ps, production runs of varying length were performed at every λ-value: 10 ns at all λ-values for the ligands free in solvent, and at least 1 ns in protein complexes, with longer simulations at selected λ-values. Unconverged simulations, as estimated by their hysteresis (see below), were prolonged up to 20 ns. If a simulation’s analysis yielded abruptly changing 〈∂H/∂λ〉_λ_ values, additional λ-points were interspersed to achieve a smooth and accurate mutation curve for thermodynamic integration. Table S2 in the Supplementary Materials gives a detailed overview of all individual simulation times per λ-point.

### 2.3. Calculation of Relative Binding Free Energy

To calculate free-energy differences between two arbitrary states, A and B, alchemical methods use a coupling or scaling parameter λ. Each state’s total energy is given by its Hamiltonian, and alchemical transformations along λ are applied to change state A gradually into state B via a number of discrete step, followed by thermodynamic integration (TI) [30]. The coupling parameter λ defines a path that connects the two Hamiltonians of state A and B, and it is introduced such that at λ = 0, the Hamiltonian H(λ) represents state A, and at λ = 1, it represents state B. At intermediate λ-values the exact functional form of H(λ) is continuous between A and B and represents intermediate states [31].

H (λ, **r**, **p**) = (1 − λ)∙H_A_ (λ, **r**, **p**) + λ∙H_B_ (λ, **r**, **p**) (1)

Variables **r** and **p** in Equation (1) refer to positions and momenta of all constituting particles, respectively.

The free-energy difference ΔG_AB_ between two states can be calculated by integration along any path from A to B, according to Equation (2) [30]:

(2) $$\Delta G_{\mathrm{mutate}}\;(A\to B)=G(B)-G(A)=\int_{0}^{1}{\langle \frac{\partial H}{\partial \lambda }\rangle }_{\lambda }d\lambda$$

This holds true irrespective of the ability to realize this path as a physical process. The term in angular brackets denotes the ensemble average of the derivative of H with respect to λ, which is obtained from independent simulations at (discrete) intermediate values of λ. A final free-energy estimate is obtained by numerical integration over this ensemble average.

In this study, states A and B each correspond to a different tripeptide ligand, and alchemical transformations are carried out separately between all three possible pairwise ligand combinations. Intermediate states between A and B represent strictly theoretical “hybrid” ligands. States A and B could also be chosen to represent the bound and unbound states of a single fixed ligand. The path connecting A and B could then be used to calculate the free energy of binding directly. It is, however, computationally unnecessarily more demanding to do so.

The more closely related states A and B are, the faster convergence will be achieved. In order to monitor convergence of the simulations used for TI, the free energy corresponding to every process was also calculated in reversed direction. This “backward” calculation starts at state B and connects via the coupling parameter λ back to state A. In practice, the final structure of the forward process (after the last equilibration step) was used as input to start the corresponding backward calculation. The difference between integration results from forward and (sign-inverted) backward calculations is called the hysteresis and should approach zero for converged simulations, since both should draw from the same ensembles.

Another way to monitor convergence of the simulations used for TI is to construct a thermodynamic cycle that connects all investigated end states (ligands) with each other through the appropriate alchemical transformations. As free energy is a state function, the sum of any changes along an alchemical path that leads back to the original state must amount to zero, independent of the path itself. Deviation of this cycle closure value from zero beyond thermal noise likely points to insufficient sampling of relevant conformations:

0 ± 2.5 kJ/mol = ΔG_mutate_ (A → G) + ΔG_mutate_ (G → S) + ΔG_mutate_ (S → A) (3)

where ΔG_mutate_ (S → A) = −ΔG_mutate_ (A → S). Figure 2 shows the thermodynamic cycle between all three ligands, applied to water and protein environment. It also illustrates how the relative free energies of binding between two ligands may be computed from two alchemical transformations between them. For example, from the cycle:

0 = ΔG_mutate,solv_ (A → S) + ΔG_bind_ (S) − ΔG_mutate,bound_ (A → S) − ΔG_bind_ (A) (4)

We obtain:

ΔΔG_bind_ (A → S) = ΔG_bind_ (S) − ΔG_bind_ (A) = ΔG_mutate,bound_ (A → S) − ΔG_mutate,solv_ (A → S) (5)

Table 1 compares the relative binding free energies (∆∆G_bind_) calculated from MD simulations to experimental values. For each transformation all available forward and backward values, as depicted in Figure S2 in the Supplementary Materials, were averaged.

## 3. Results and Discussion

### 3.1. TI of Ligands Free in Solvent

#### 3.1.1. Free-Energy Convergence

As a starting point, all TI calculations were performed by evaluating trajectories of 1 ns length per λ-point, resulting in a total of 11 ns per transformation run. This lead to hystereses of 0.1, 0.1, and 1.2 kJ·mol^−1^ for the KGK → KAK, KGK → KSK, and KAK → KSK transformations, respectively, when employing the increased softness parameter (α = 1). The thermodynamic cycle closed with a value of 0.3 ± 1.5 kJ·mol^−1^. After prolongation to 10 ns per λ-point for all transformations, every individual hysteresis was reduced to 0.3 kJ·mol^−1^ or below, and cycle closure improved to 0.2 ± 0.8 kJ·mol^−1^.

**Figure 3:**
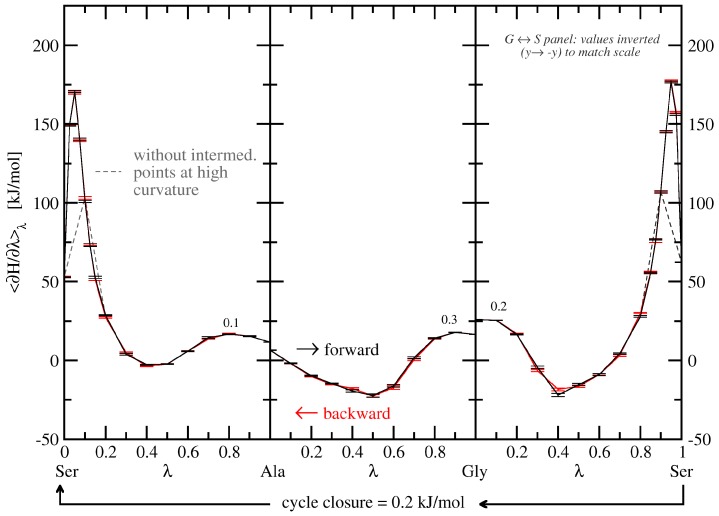
Final thermodynamic cycle in H_2_O, at 10 ns simulation time per λ-point for all TI curves. Solid black lines are forward transformations (read from left to right), solid red lines are backward transformations (read from right to left; shown with inverted sign and direction for better comparability to the forward process). Dashed lines are provided to highlight the necessity of additional λ-values at areas of high curvature. Note that for the G ↔ S transformations the negative value of 〈∂H/∂λ〉_λ_ is plotted to match the scale. Numbers directly above and below the lines refer to the hysteresis between associated forward and backward transformations in in kJ·mol^−1^.

The values of 〈∂H/∂λ〉_λ_ as a function of λ (free-energy profile/TI curve) for the three TI processes free in solvent are shown in Figure 3. The corresponding numerically-integrated values and their averages are given in Table 1.

#### 3.1.2. Dihedral Angles

**Figure 4:**
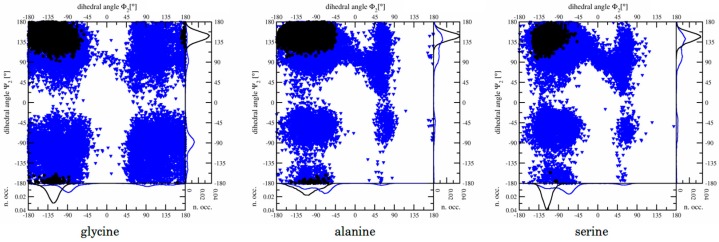
Narrowing of sampled conformational space upon binding, monitored via central side chain dihedral angles. Blue triangles: free in solvent; black circles: complexed in OppA. Data was extracted from the longest available simulation of the end state at λ = 0, (*i.e.*, the crystal structure before alchemical changes): All ϕ_2_/ψ_2_ side chain dihedral angles are monitored over 10 ns simulation time, except for KSK complexed in OppA (right panel, black circles), for which only 3 ns simulation time was available. Distributions are summed up for each angle in the flanking graphs on the right and below each Ramachandran plot, and the number of occurrences (n.occ.) was normalized.

Among the three peptide ligands, conformational freedom is significantly larger for KGK than for KAK and KSK, as is illustrated in Figure 4. Surprisingly, the minimal chemical difference of a single methyl group between KGK and KAK resulted in a very poor hysteresis between forward and backward simulations in preliminary studies (with α = 0.5). It turned out that sampling of the ϕ_2_- and ψ_2_-angles (describing the peptide backbone geometry at the central, mutated residue, see Table S1 in the Supplementary Materials) was particularly inefficient for this transformation. The difference in ϕ_2_/ψ_2_ dihedral angle distribution occurring at corresponding λ-values (identical intermediate system states) along forward and backward transformation processes was identified as the origin of the remarkably large hysteresis observed in this case. While the differences in conformational freedom between the peptides is physically well-known, slow transitions between the different conformations hamper an efficient free energy calculation.

**Figure 5:**
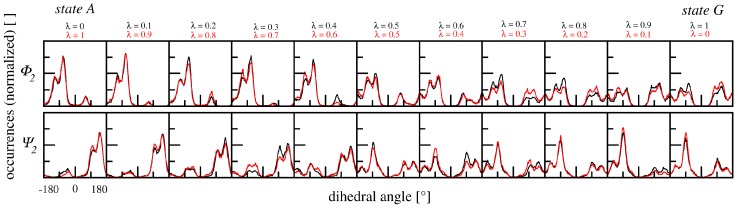
Dihedral angle distribution for KAK → KGK along forward (black) and backward (red) transformations in solvent. ϕ_2_/ψ_2_ angles are monitored over a 10 ns simulation trajectory at each λ-point. Forward and backward transformations are in agreement for both angles. Additionally, distributions show clear peaks for the side chain bearing state A, and wider fluctuations for state G which lacks a side chain.

Figure 5 shows the distributions of ϕ_2_- and ψ_2_-angles along the transformation process in the system at hand (using α = 1.0) and demonstrates agreement between forward and backward transformations with sufficient sampling time, in agreement with the observed small hysteresis of the TI curves in Figure 3.

### 3.2. TI of Ligands Bound to the Protein

**Figure 6:**
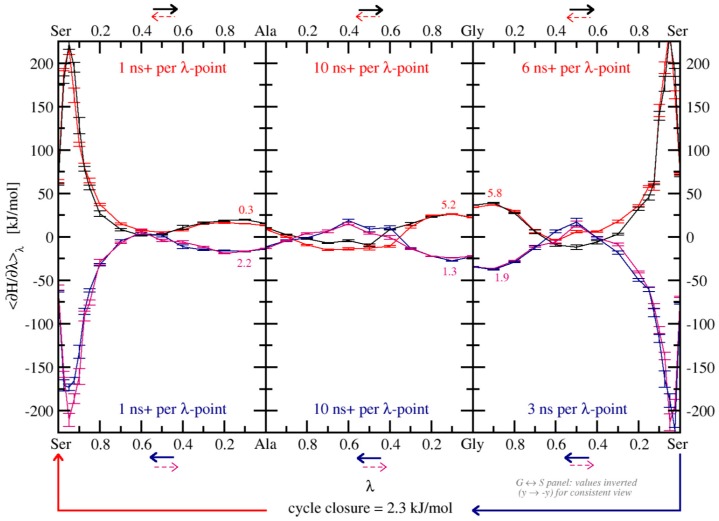
Final thermodynamic cycle in OppA. Black and blue lines are forward transformations, red and purple lines are backward transformations (reading direction according to the arrows above resp. below each panel). Like in Figure 3, backward TI curves are shown with inverted sign and direction for better comparability to the forward process. The minimum trajectory length for all λ-points of a given panel is written in the respective color on the top and bottom of that panel. See Table S3 in the Supplementary Materials for individual prolongation times of diverging λ-points. Numbers directly above and below the lines are colored to refer to the hysteresis between associated forward and backward transformations and are given in kJ·mol^−1^.

In a next step, the same alchemical transformations as in water were carried out with the complexed ligands in protein environment. The resulting free-energy profiles are depicted in Figure 6.

#### 3.2.1. Free-Energy Convergence

Since initial TI calculations with a simulation time of 1 ns per λ-point showed poor convergence, most TI calculations for the OppA environment depicted in Figure 6 had to be prolonged until a cycle closure below thermal noise could be reached (final value 2.3 ± 2.8 kJ·mol^−1^). Table S2 in the Supplementary Materials gives an overview about added λ-points and individual simulation times per λ-point. A large hysteresis (up to 8 kJ·mol^−1^) remained especially for KAK → KGK and KGK → KSK in complex with OppA even at simulation lengths of up to 10 ns per λ-point, making prolongations of up to 20 ns per λ-point necessary for these transformation runs. Overall, Figure 6 depicts a total of about 1.3 µs of atomistic simulation time. The integrated values of 〈∂H/∂λ〉_λ_ as a function of λ for all forward and backward TI processes in Figure 6 are shown in Table 1.

#### 3.2.2. Energy Contribution Analysis

**Figure 7:**
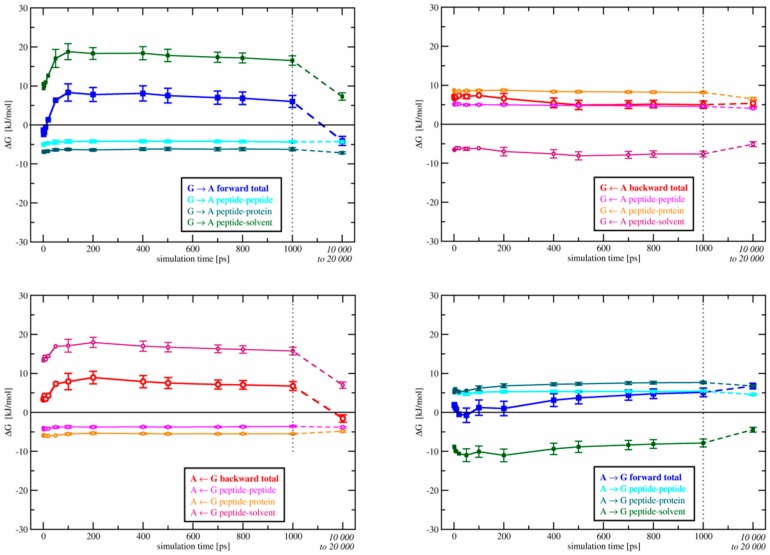
Convergence of individual contributions to the free energy for the transformations between KGK and KAK in OppA. Upper left panel: KGK → KAK forward; upper right panel: KGK → KAK backward; lower right panel: KAK → KGK forward; lower left panel: KAK → KGK backward. Contributions to the free energy are separated for peptide-peptide (intra-peptide), peptide-protein, and peptide-solvent interactions as a function of simulation time per λ-point.

Figure 7 shows the development of free-energy differences as a function of simulation time per λ-point for all transformations between KGK and KAK. The contributions due to peptide-peptide, peptide-protein, and peptide-solvent interactions are indicated separately. It is clear that the large change in free energy after simulation times longer than 1 ns is almost entirely due to peptide-solvent contributions, pointing at solvent interactions as a cause for the remaining hysteresis. Thus, the poor hysteresis of KAK → KGK transformations is likely caused by inaccurate solvation of the active site during the alchemical process. As solvation is thought to be crucial to the binding thermodynamics of OppA, an error there would give rise to a wrong free-energy estimate.

An analysis of the differences in free energy as a function of simulation time, split up into peptide-peptide, peptide-protein, and peptide-solvent contributions, has also been performed for all other transformations.

In transformations between KGK and KSK, peptide-protein interaction energy is the contribution fastest to converge and, thus, the least problematic. The contribution due to peptide-peptide interactions converges with approximately the same speed as peptide-solvent interaction energy and is, thus, comparatively slower than in the KGK → KAK case. This is to be expected since transformations between KGK and KSK represent the largest investigated chemical change to the ligand, *i.e.*, growth of a whole hydroxymethyl group. However, free-energy convergence is less problematic in general for all of the KGK → KSK transformations as compared to the KGK → KAK transformations, pointing out again the special nature of the latter case.

For the transformations between KAK and KSK, free-energy convergence was comparatively fast; thus, no individual contribution was distinguishable as problematic.

Over all transformations, the contribution of peptide-protein interaction energy seems to converge towards approximately the same value of ~8 kJ·mol^−1^; the final values of peptide-peptide and peptide-solvent interactions vary over a much wider range. This confirms that the appearance of a polar side chain in position two is not contributing directly to OppA’s ligand discrimination.

#### 3.2.3. Dihedral Angles

Figure 4 shows that conformational sampling of all ligands in the bound state is tightly restricted in comparison to the free state. This loss of conformational freedom and the associated reduction in conformational entropy is largest for KGK and explains its weak binding affinity [14,15,18]. Some methods regard conformational entropy loss as constant for all tripeptide ligands of the KXK series [16]. However, from Figure 4 it seems unlikely that the cause of the observed hysteresis in KGK transformations would be the flexibility of the glycine residue. It is probably rather another characteristic of the KGK crystal structure: abundance of water in the binding site.

#### 3.2.4. Solvent Network Rearrangement

**Figure 8:**
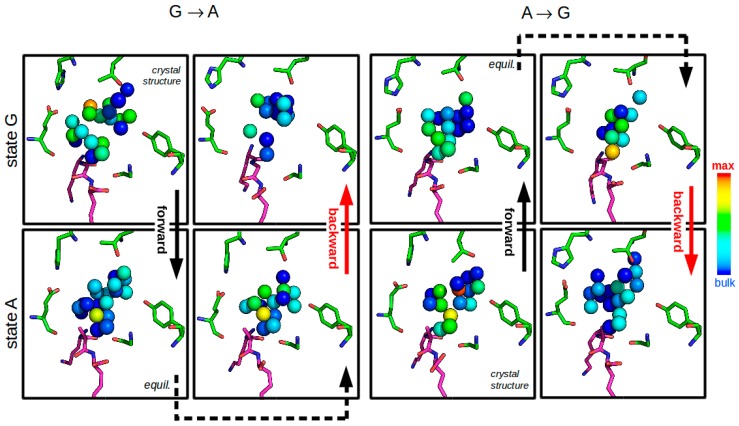
Depiction of average water occurrences over the 10 ns end-point TI simulations of transformations between KGK and KAK. Protein and ligand atoms are depicted at their average positions after a superposition based on the backbone atoms. The average water occurrence frequency was monitored on a grid with a spacing of 0.2 nm. Water molecules are assigned to their nearest grid point. Grid points with at least 30% occupancy of the highest observed occupancy (corresponding to bulk water) are indicated in the figure and colored accordingly (red ~90%; green ~50%; dark blue ~30%). For clarity, some water locations close to the lysine-1 and lysine-3 side chains of the ligand have been removed. Panels in the same row represent identical system states. Note the lowered water densities when comparing the first and the last panel of each transformation run (upper left two for KGK_crystal_ → KGK_final_, lower right two for KAK_crystal_ → KAK_final_).

Changes in solvation of the S2 binding pocket are visualized in Figure 8, which monitors water molecules in the active site during end point simulations of the transformation processes between KGK and KAK. The occurrence of water molecules was measured on a grid with a spacing of 0.2 nm, and each water molecule was assigned to its nearest grid point. In the forward transformation process KGK_crystal_ → KAK, the emerging alanine side chain indeed seems to restrict the space available to the water molecules, resulting in a specific pattern of lowered water occurrence. However, in the backward process, the number of occurrences per grid point is clearly not being restored to its original amount. Comparing KGK_crystal_ to KGK_final_, it can be seen that the water molecules do not refill the gap between glycine and the protein after the backward transformation is completed. Their diffusion is simply too slow for the effective transformation time.

The lagging adaption of the water network to the emerging (and, to an even larger extent, the vanishing) hydrophobic side chain presumably is responsible for slow convergence of the peptide-solvent interaction energy. This is reflected in the hysteresis of this transformation, which improved from 11 to ~1 kJ·mol^−1^ after prolongation from 11 to 140 ns overall simulation time for each of the forward and backward transformation runs.

According to Figure 7, the same slow convergence is causing the hysteresis in the KAK_crystal_ → KGK transformation, which however was only reduced from 12 to 5 kJ·mol^−1^ after prolongation from 11 to 160 ns. This suggests that significantly longer simulations may at some point overcome this sampling problem. However, it should be noted that this will be necessary for all diverging intermediate λ-values in both the forward and backward calculations, ideally for all transformations suffering from slow adaption of water. Roughly estimated, an additional 100–150 ns of simulation time could bring the remaining hysteresis down to about 1 to 2 kJ·mol^−1^ for transformations between KGK and KAK (plus additional ~100 ns for transformations between KGK and KSK for the same result on the other remaining hysteresis).

### 3.3. Relative Binding Free Energies

In spite of the remaining hysteresis, the average of all TI values corresponding to a given alchemical processes were calculated [32]. By comparing these averages between transformations conducted freely in solvent *versus* complexed in the protein, relative binding free energies can be calculated. These are compared to the experimental values in Table 1.

The individual cycles close within thermal noise and amount to zero within their error ranges. The total cycle adds up both the solvent and protein environment transformations. Consequently, the total cycle closes around zero within its summed up errors as well, and in addition, the final result of 2.5 ± 2.9 kJ·mol^−1^ also closes to zero within thermal noise.

The TI prediction for KGK → KAK is in excellent agreement with experiment, with an error of merely 1.0 kJ·mol^−1^. KGK → KSK and KAK → KSK are reasonably close to experimental values—within 3.8 kJ·mol^−1^ (less than 1 kcal·mol^−1^) and 5.4 kJ·mol^−1^ (order 10 in K_d_), respectively—and the qualitative order of relative binding affinities is very clearly reproduced.

## 4. Conclusions

Much valuable information on the thermodynamic properties and structural features of oligopeptides binding to OppA is available, shedding light on the molecular recognition process. This system represents an opportunity to specifically study the role of water in the binding of chemically-diverse ligands because OppA provides a constant protein structure.

In this study, only minimal alchemical changes were pursued: the transformation of a KAK to a KGK ligand, for example, involves only a single methyl group. The large conformational flexibility of glycine affects convergence of the TI calculations in water environment considerably. By employing increased soft-core potentials this challenge could be overcome, smoothing the transition from side chain-less to side chain bearing amino acids.

However, while this modification could solve the large discrepancy in freely solvated environment, the TI profiles obtained for the protein complexes remained unconverged, initially. The cause of these discrepancies seems to lie with the slow dynamics of the water molecules. Water occurrence in the subpocket of interest in forward *vs.* backward transformation processes was shown to display different patterns for the same system states. By significantly prolonging simulation times and giving water time to adapt to the alchemical changes, thermodynamic cycle closures within error range and thermal noise could finally be achieved, however. Further improvements in the methodology could include enhanced sampling through Hamiltonian replica exchange simulations, the simultaneous removal of a water molecule from the active site, or a combination of these approaches. We will investigate these possibilities in future work.

Overall, the resulting relative binding free energies of the compounds KAK, KGK, and KSK bound to the OppA protein were in good agreement with experimentally determined values. This work demonstrates the challenges in quantitatively describing the promiscuous water-mediated binding of ligands to a common protein host.

## Figures and Tables

**Table 1:** Comparison of values for ΔG_mutate_ and ΔΔG_bind_, in kJ·mol^−1^.

Alchemical Mutation	ΔG_mutate,solv_	ΔG_mutate,bound_	ΔΔG_bind,calc_	ΔΔG_bind,exp_
A → G	−3.9 ± 0.4	6.8 ± 0.7		
A ← G	4.2 ± 0.4	−1.6 ± 1.0		
G → A		−4.1 ± 1.1		
G ← A		5.4 ± 0.9		
average A → G	−4.0 ± 0.4	4.4 ± 0.9	8.5 ± 1.0	7.5 ± 2.4
G → S	−22.5 ± 0.5	−31.6 ± 1.9		
G ← S	22.4 ± 0.5	37.4 ± 1.7		
S → G		−35.7 ± 2.2		
S ← G		−33.8 ± 2.0		
average G → S	−22.4 ± 0.5	−34.6 ± 2.0	−12.2 ± 2.0	−8.4 ± 0.9
A → S	−26.2 ± 0.4	−30.2 ± 1.7		
A ← S	26.3 ± 0.4	32.4 ± 2.0		
S → A		33.5 ± 1.9		
S ← A		−33.9 ± 1.4		
average A → S	−26.3 ± 0.4	−32.5 ± 1.7	−6.3 ± 1.8	−0.9 ± 2.2
cycle	−0.2 ± 0.8	2.3 ± 2.8	2.5 ± 2.9	0

Individual estimates of ΔG_mutate_ were obtained by integration of the respective TI curves. Values for ΔΔG_bind_ were calculated from the differences between the averages over all available forward (black) and backward (red) transformations, as depicted in Figure S2. Experimental values are taken from reference [15], and error estimates were calculated from these data. Experimental cycle closure is 0 by definition. For clarity, only the mutated central residue of the tripeptide ligand is pointed out.

## Supplementary Materials

Supplementary materials can be accessed at: http://www.mdpi.com/1420-3049/21/4/499/s1.

## Abbreviations

The following abbreviations are used in this manuscript:

MD	molecular dynamics
n.occ.	number of occurrences
OppA	oligopeptide-binding protein A
QSAR	quantitative structure-activity-relationship
TI	thermodynamic integration
