# Supplementary material for: Calculation of Relative Binding Free Energy in the Water-Filled Active Site of Oligopeptide-Binding Protein A
Source: Molecules. 2016 Apr 15;21(4):499. doi: 10.3390/molecules21040499 (PMC5881882; doi:10.3390/molecules21040499)
Supplement: Supplementary file 1 [file molecules-21-00499-s001.pdf]

Supplementary Materials: Calculation of relative binding free energy in the water-filled active site of oligopeptide-binding protein A

Manuela Maurer, Stephanie B.A. de Beer and Chris Oostenbrink

Table of Contents

Figure S1	Superposition of the investigated crystal structures, depicting the S2 pocket which hosts the central residue of KXK.
Figure S2	Practical implementation of both thermodynamic cycles in the OppA system, depicting the overall simulation scheme.
Figure S3	Detailed scheme of a single MD simulation run along the coupling parameter l for an alchemical transformation.
Table S1	Structural details of the tripeptide ligands constructed for alchemical transformation, and their experimentally determined binding free energies to OppA in kJ mol-1.
Table S2	Length of simulation trajectories at individual l-points, in ns, listed for all transformation runs in OppA.

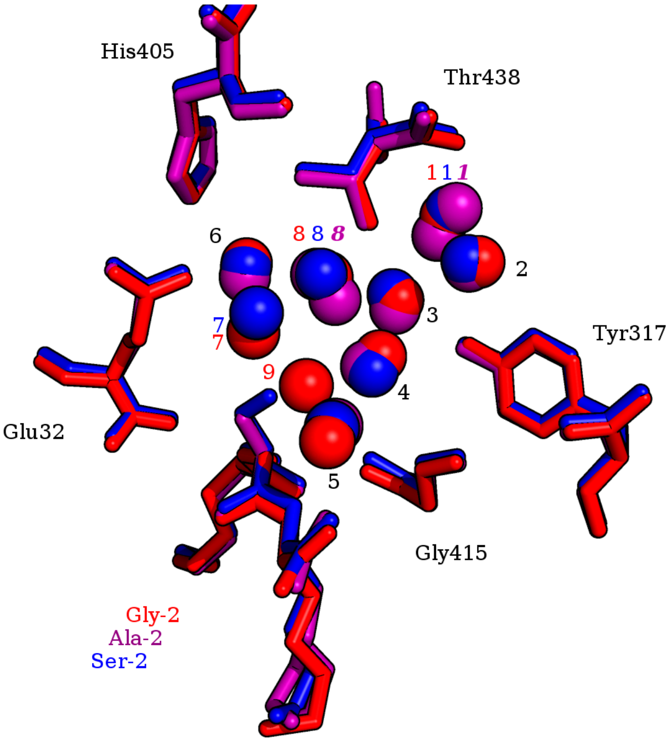

Figure S1. Superposition of the investigated crystal structures, depicting the S2 pocket which hosts the central residue of KXK. Water numbering according to reference [15]. Crystallographic waters are depicted as spheres. Red: 1B3L (X = glycine). Purple: 1JET (X = alanine). Blue: 1B51 (X = serine). Water number 9 is expelled by alanine and serine side chains. Water number 7 is expelled only by the hydrophobic alanine side chain, but retained by the larger polar serine side chain. Note how only alanine causes discrete disordering of the solvent network, as evidenced by water molecules number 1 and 8 (italic) with lower crystallographic occupancy (visible here as purple overlapping spheres) next to Thr438.

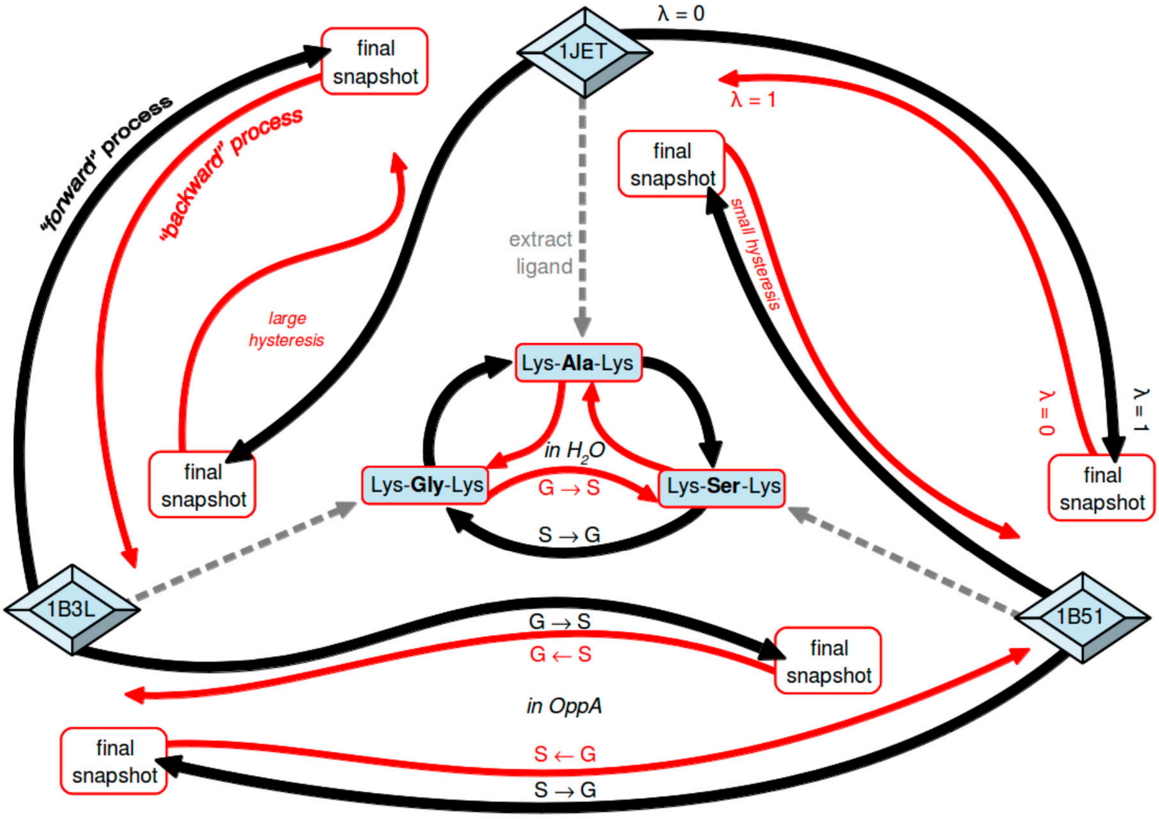

Figure S2. Practical implementation of both thermodynamic cycles in the OppA system, depicting the overall simulation scheme. Black arrows and blue fields correspond to forward simulations, initiating from the respective crystal structures. Red arrows and frames correspond to backward calculations, using output of a completed forward transformation as the initial structure. Distance between arrows represents hysteresis. Process naming conventions are given using the example of transformations between glycine and serine.

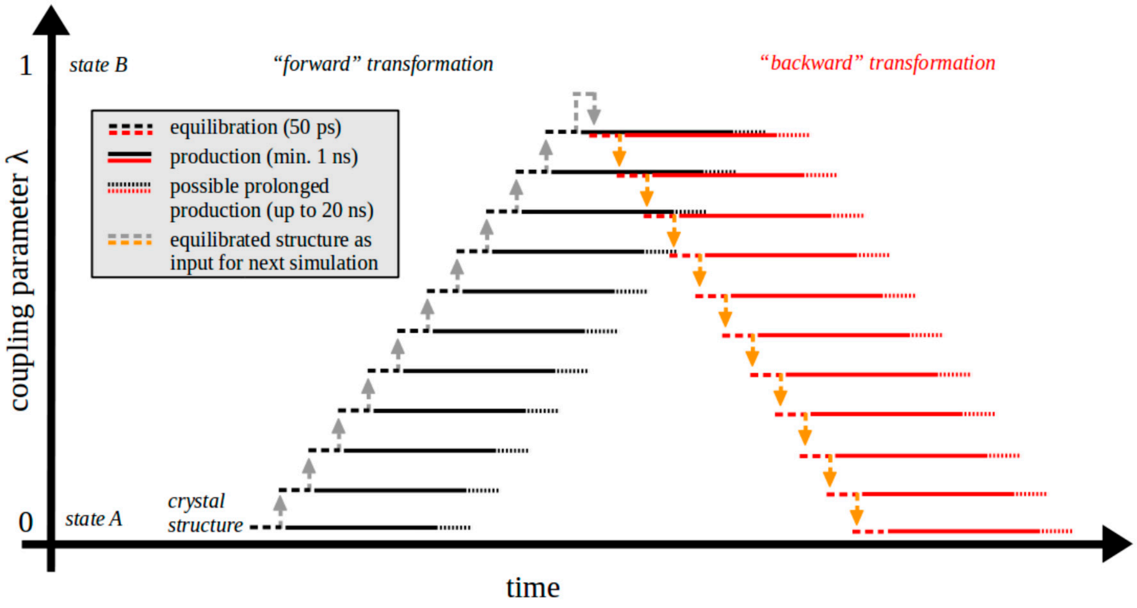

Figure S3. Detailed scheme of a single MD simulation run along the coupling parameter λ for an alchemical transformation.

Table S1. Structural details of the tripeptide ligands constructed for alchemical transformation, and their experimentally determined ([a] references [14,15]) binding free energies to OppA in kJ mol^{-1}, with error estimates calculated from these data. Outlined letters refer to atoms for which the Lennard-Jones potential and electrostatic interactions are turned off (dummies).

Alchemical Structure	Tripeptide Ligand (KXK)	PdB Code	$\Delta G_{\text{bind, exp}}$[a]
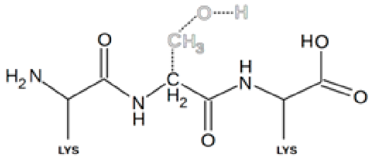	Lys-Gly-Lys (KGK)	1B3L	−33.6 ± 0.9
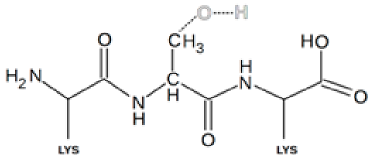	Lys-Ala-Lys (KAK)	1JET	−41.1 ± 0.3
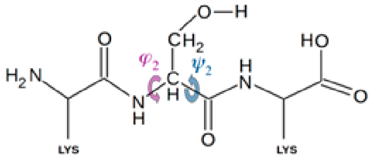	Lys-Ser-Lys (KSK)	1B51	−42.0 ± 2.2

Table S2. Length of simulation trajectories at individual λ-points, in ns, listed for all transformation runs in OppA. Black denotes forward transformations, red denotes backward transformations.

λ forw.	0.000	0.025	0.050	0.075	0.100	0.125	0.150	0.200	0.300	0.400	0.500	0.600	0.700	0.800	0.850	0.875	0.900	0.925	0.950	0.975	1.000
λ backw.	1.000	0.975	0.950	0.925	0.900	0.875	0.850	0.800	0.700	0.600	0.500	0.400	0.300	0.200	0.150	0.125	0.100	0.075	0.050	0.025	0.000
A → G	10				10			20	20	20	20	20	10	10			10				10
A ← G	10				10			20	20	20	20	20	10	10			10				10
G → A	10				10			10	10	20	20	20	10	10			10				10
G ← A	10				10			10	10	20	20	20	10	10			10				10
G → S	6				6			6	6	20	20	20	20	20	20	20	6	6	6	6	6
G ← S	6				6			6	6	20	20	20	20	20	20	20	6	6	6	6	6
S → G	3	3	3	3	3	3	3	3	3	3	3	3	3	3			3				3
S ← G	3	3	3	3	3	3	3	3	3	3	3	3	3	3			3				3
A → S	1				1			1	1	1	1	1	5	5	10	10	10	10	10	10	1
A ← S	1				1			1	1	1	1	1	5	5	10	10	10	10	10	10	1
S → A	1	3	3	3	3	3	3	1	1	1	1	1	1	1			1				1
S ← A	1	3	3	3	3	3	3	1	1	1	1	1	1	1			1				1
